# Supplementary material for: Using Wash’Em to Design Handwashing Programmes for Crisis-Affected Populations in Zimbabwe: A Process Evaluation
Source: Int J Environ Res Public Health. 2024 Feb 23;21(3):260. doi: 10.3390/ijerph21030260 (PMC10970461; doi:10.3390/ijerph21030260)
Supplement: Supplementary file 1 [file ijerph-21-00260-s001.zip › S1. Document_Rapid Assessment tool guide_Handwashing Demonstrations.pdf]

# Handwashing Demonstrations

**Guide**

## Purpose

**This tool allows you to generate quick insights into whether a person's home and community environment enable or prevent handwashing practices. It is useful to start with this tool as it requires you to go to people's homes and understand how handwashing is performed in a real-world setting. During the demonstrations, you should note how people interact with objects (for example, soap and containers) and infrastructure (for example, handwashing facilities and water points) when handwashing. You should also look out for hesitations during the demonstration as this may indicate that handwashing is not practiced regularly. Pay attention to what makes the process inconvenient or undesirable. There is evidence to suggest if we can understand the settings where the target behaviour takes place, then we can predict how people will behave with 90% accuracy. By following this guide, you will learn which things to look out for so you can tell whether handwashing is a part of normal routines. You will learn how to identify barriers that might prevent behaviour and design interventions that make handwashing more highly prioritized, more convenient, and more desirable.**

## Requirements

### Time

5–10 minutes per participant

### Format

A minimum of 06 individual observations

### Participants

Select individuals so that your sample includes a mix of ages, gender, abilities, ethnicities, and religions. Also, select your participants based on diversity of location, proximity to water sources, and access to resources. For more information about how to choose participants, read the separate *Selecting Participants* guide in the *Quick Tips* section of the website.

### Materials

- Video recording device (for example, mobile phone or camera with video recording function)
- Handwashing Demonstration consent script (at the end of the guide)
- Handwashing Demonstration decision making table (separate Excel sheet)

### Roles

- *Facilitator:* One person to talk with the participant to explain the process
- *Video recorder:* One person to record the demonstration
- *Analysis team:* Several team members to analyze and discuss the video-recorded demonstrations

## Data Collection

### Preparation

- Check that the recording device works and has sufficient battery life.

- Practice a demonstration.
- Watch the example videos provided in the training guide so you become more familiar with how to identify barriers that might prevent handwashing.
- Prepare a consent script in the local language.

## Consent

Ask the participants for their consent:

- Introduce yourself.
- Explain what you are doing and why.
- Briefly describe the activity.
- Tell the participants how you will use their information.
- Assure them of confidentiality.
- Ask participants if they are willing to participate.
- Explain there will be no consequences if they do not wish to participate.

See a sample consent request script at the end of this guide.

## Activity

1. Decide whether you want to learn about handwashing after toilet use or handwashing before food preparation and eating. If you want to learn about both, then you will need to do six videos at each location. If you are limited with time, focus just on handwashing after using the toilet.
2. Ask the participant to show you how they normally wash their hands, either after using the toilet, or before eating. Be sure not to mention soap, as doing so could bias your results. Make it clear they should demonstrate handwashing in their normal location (for example, at their own home or near the toilet they usually use), using the products and procedures they normally use. If necessary, walk with the participant to the location where handwashing normally takes place. Be aware that this is a strange thing to ask of someone. Often, people will laugh at the request. You may want to clarify that you are not actually asking them to use the toilet or prepare food.

### Tips

- Try to get a mid-shot *(see Figure 1)* with the participant's hands at the centre; this will make it easier to keep the person anonymous when the video is reviewed.
- Try to remain neutral (for example, resist the urge to speak or provide guidance).

***Figure 1***
Mid-shot of person washing hands

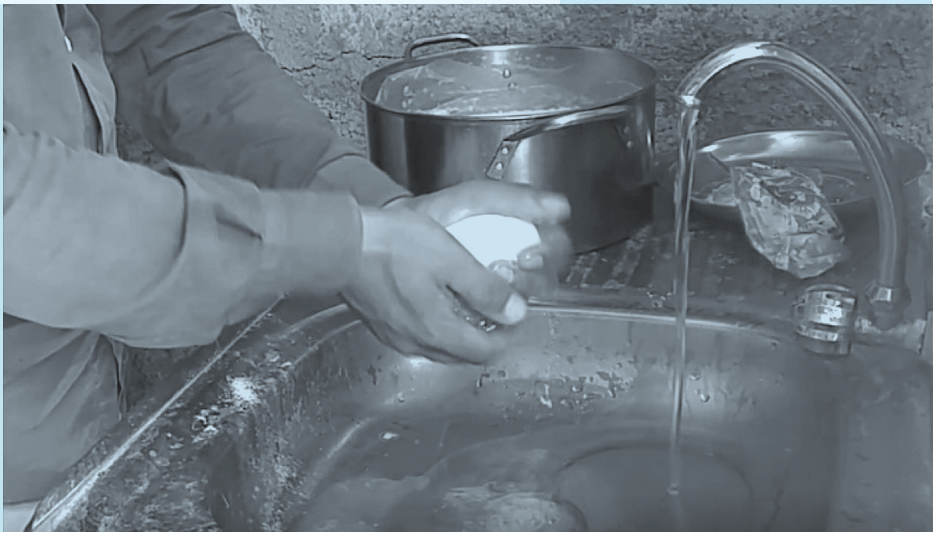

*Source: Sian White. Used with permission.*

3 Remind the participant you will be videotaping them, but you will try not to get their face in the video.

4 Start the video recording as soon as the person gets up and moves toward the toilet or kitchen. Timing is important because you can make observations on the way to the handwashing location. Record for the duration of the handwashing process even if it means following the participant to different locations.

5 Show the participant the video you took.

6 Thank the participants for their time.

## Analysis

1 When you return to the office, open the project you have created in the Wash'Em Program Designer.

2 Re-watch the video recordings you took and follow the instructions as you enter the data into the Wash'Em Program Designer.

3 The Program designer will highlight key patterns emerging from your data. Read the section analysis to learn how to interpret these results and which behaviour change challenges to focus on.

## Recommendations

1 After entering the results from all the tools you've used, click on the Generate Recommendations button in the Program Designer.

2 As a team, discuss the recommendations and make a plan to implement them.

## Tool Limitations

During the handwashing demonstrations, participants may want to impress you. Participants may show you what they think you want to see rather than what they normally do. **You may observe that they spend more time and care than normal when washing their hands.** You need to look for any hesitations during the demonstration, as well as what makes the process inconvenient, uncomfortable, or not feasible. These nuances are what you learn most from.

## Handwashing Demonstrations Consent Script

It is important your participants are provided with appropriate details about why you are collecting information from them, what will be required of them, and how the information will be used. When using the Handwashing Demonstrations tool, you can use the following explanation:

> Hi, my name is________and I work for________organization. We are visiting your community/camp to learn more about people's lives and behaviours here. Today, we are particularly interested to learn about handwashing practices. If you are willing to help us, then we would like you to demonstrate how you normally wash your hands. As you do the demonstration, we would like to videotape you and take notes. We will make sure not to get your face in the video, and we will not take your name; this means we will protect your identity. You can also view the video when we are done. The video will only be viewed by people in our organization and will not be shared more broadly. (It will not appear on social media or TV). We are not here to judge your behaviour, just to learn from you. The things we learn will be used to design programs to help people in communities/camps like yours. There are no consequences to you or your family if you do not wish to participate. Do you wish to participate?

20.02.01



USAID FROM THE AMERICAN PEOPLE
ACTION CONTRE LA FAIM
LONDON SCHOOL of HYGIENE &TROPICAL MEDICINE
CAWST

This guide was made possible by the generous support of the American people through the United States Agency for International Development (USAID).
The contents are the responsibility of LSHTM, ACF and CAWST and do not necessarily reflect the views of USAID or the United States Government
