# Supplementary material for: Using Wash’Em to Design Handwashing Programmes for Crisis-Affected Populations in Zimbabwe: A Process Evaluation
Source: Int J Environ Res Public Health. 2024 Feb 23;21(3):260. doi: 10.3390/ijerph21030260 (PMC10970461; doi:10.3390/ijerph21030260)
Supplement: Supplementary file 1 [file ijerph-21-00260-s001.zip › S2. Document_Rapid Assessment tool guide_Motives.pdf]

Motives
Guide

Purpose

Educating people about why they should wash their hands with soap rarely gets them to do it. That's because there are many factors that affect the way our brains makes decisions. Motives drive humans to do things that meet their short-term goals (such as putting on a coat if we are feeling cold) and their long-term goals (such as completing many years of education because we believe that knowledge can improve our status within society). Everyone has individual goals and dreams. But the motives that drive these goals and dreams are shared by all humans, irrespective of circumstances, culture, or beliefs. The reason we share these motives is because, throughout human history, we have passed on skills and behaviours that help us to live longer, more enjoyable, and more productive lives. The Motives tool will help you to identify what is driving handwashing behaviour, or preventing it, in your context. It will also identify motives that shape people's identity and other behaviours. You will use these motives to develop a motivational story for your handwashing intervention.

Requirements

Time
20-40 minutes per focus group discussion

Format
A minimum of three focus group discussions (FGDs)

Participants
Form one focus group with approximately seven women and conduct a separate one with seven men.

> **Tip:** Try to make sure that the people in each FGD are similar. Mixing men and women, or young people and older people can affect power dynamics and make some participants reluctant to speak.

Materials
- Motives character cards (at the end of the guide)
- Motives consent script (at the end of the guide)
- Motives decision making table (separate Excel sheet)

Roles
- *Facilitator:* One person to facilitate the activity and discussion
- *Scribe:* One person to fill in the worksheet to capture key information from the participants
- *Analysis team:* Several team members to analyze and discuss the findings

Data Collection

Preparation

- The *character cards* should be translated into the local language in the blank space provided.
- Print and cut out each of the *character cards*.
- Print the Motives worksheet.
- Read the separate *Tips for Effective Focus Group Facilitation* guide in the *Quick Tips* section of the website.
- Rehearse the activity with a team member.

Consent

Ask the participants for their consent:

- Introduce yourself.
- Explain what you are doing and why.
- Briefly describe the activity.
- Tell participants how you will use their information.
- Assure them of confidentiality.
- Explain that there will be no consequences if they do not wish to participate.
- Ask participants if they are willing to participate.
  (See a sample consent request script at the end of this guide.)

Activity

1. Locate an appropriate space to conduct the FGD. Try to find somewhere that is private and conveniently located for the participants.
2. Outline the rules for the FGD to participants, explaining there are no right or wrong answers to anything they are asked. Ask them to be respectful of all opinions and not to interrupt or talk over one another. To encourage everyone to share freely, request that they do not share what was discussed with anyone outside of the group after the session.
3. Briefly introduce each of the characters on the character cards and explain what each card is showing. Then, put each card down in the middle of the focus group circle. The images on each card are designed to represent a character, but may not be obvious without explanation. It is your job to fully explain the character to the participants by reading the small print written on each card. Do not ask the participants to tell you what they think the character is, as doing so could create confusion.
4. When all the cards are spread out on the table, check that the participants remember the characters. Show one card at a time and ask participants, “What is this character?” This step is particularly important for participants who cannot read or write.
5. Ask the participants to rank the character cards from the person they think is most likely to always wash their hands with soap to the person who they think is least likely to practice handwashing with soap. Make sure everyone is involved in developing the ranking order.
6. Once the order is finalized, go through each card and ask why they ranked each character where they did.

7 Wrap up the activity by asking two additional questions:
- Which of the characters would you aspire to be like?
- Which character do they currently feel most like?

For both of these questions make note of the two most commonly mentioned characters. You may need to get people to vote for their preference.

8 Thank the participants for their time.

Scribe

1 Record the final ranking of the characters on the Motives worksheet. You may want to take notes on any interesting comments the group makes.

2 For the last two questions, write down the two most commonly mentioned characters for each question.

Analysis

1 Discuss with your colleagues any surprising opinions that emerged from the discussion.

2 When you return to the office, open the project you have created in the Wash'Em Program Designer. Follow the instructions as you enter the ranking and your results from each of your focus groups into the Wash'Em Program Designer.

3 The Wash'Em Program Designer will highlight key patterns emerging from your data. Read the section analysis to learn how to interpret these results and which behaviour change challenges to focus on.

4 If you encounter a Section Conflict and are unable to carry out more FGDs, to help you resolve the conflict, go back to your completed worksheets and look to see which of the options available in Q31 feature highest in the ranking carried out in the FGDs. For example, if *A person who is wealthy and has a nice house* was placed higher than *A person who is upset*, select this option. It is important to go back to the collected data to help you resolve this conflict, rather than using your own judgement or "common sense" – we need to make sure that the selections we make our based on the data we have collected where possible

Recommendations

1 After entering the results from all the tools you've used, click on the Generate Recommendations button in the Wash'Em Program Designer.

2 As a team, discuss the recommendations and how you plan to implement them.

3 Discuss how you can incorporate what you learned from the Motives tool in the motivational story. For example, you may have discovered the characters most likely to wash their hands were

   a someone who is respected because of their politeness,
   b someone who is respected because of their education or wisdom, or
   c someone who values having lots of friends.

Based on this finding you could develop a motivational story that utilizes or heightens these motives.

Sample Motivational Story
A young man is leaving the toilet and forgets to wash his hands. He sees the village leader passing by and runs over to shake her hand, because handshaking is a way of showing respect in his culture. However, the village leader laughs and recoils in disgust. The young man is confused. But then, out of the corner of his eye, he sees another man leaving the toilet. The other man washes his hands thoroughly with soap as he leaves the facility and even stops to breathe in the fresh perfumed smell of his hands. Seeing the village leader, he too walks over to greet her. This time, the village leader smiles and welcomes the man warmly. "Ahh!" thinks the young man, "How foolish of me not to wash my hands with soap. Shaking hands when your hands are dirty is disrespectful."

Tool Limitations

This exercise relies on stereotypes of people. Real people do not have only one characteristic. Dealing with an abstract character may also be confusing for participants. To reduce confusion, make sure you clearly explain what each character card is trying to depict.

Motives Consent Script

It is important your participants are provided with appropriate details about why you are collecting information from them, what will be required of them, and how the information will be used. When using the Motives tool, you can use the following explanation:

> Hi, my name is________and I work for________organization. We are visiting your community/camp to learn more about people's lives and behaviours here. If you are willing to help us, we would like you to be part of a group discussion where we will ask you and six others from your community about how this emergency (for example, displacement or a cholera outbreak) has affected the lives of people around here. The discussion will involve the use of picture cards and scenarios that we would like your opinions on. During the discussion, we will take notes. People in our organization will use the information. We will not share your information more broadly. We are not here to judge you, but to learn from you. The things we learn will be used to design programs that will help people in communities/camps like yours. There are no consequences to you or your family if you choose not to participate. Do you wish to participate?

USAID FROM THE AMERICAN PEOPLE
ACTION CONTRE LA FAIM
LONDON SCHOOL of HYGIENE &TROPICAL MEDICINE
CAWST

This guide was made possible by the generous support of the American people through the United States Agency for International Development (USAID).
The contents are the responsibility of LSHTM, ACF and CAWST and do not necessarily reflect the views of USAID or the United States Government
