# Supplementary material for: Using Wash’Em to Design Handwashing Programmes for Crisis-Affected Populations in Zimbabwe: A Process Evaluation
Source: Int J Environ Res Public Health. 2024 Feb 23;21(3):260. doi: 10.3390/ijerph21030260 (PMC10970461; doi:10.3390/ijerph21030260)
Supplement: Supplementary file 1 [file ijerph-21-00260-s001.zip › S3. Document_Rapid Assessment Tool Guide_Disease Perception.pdf]

Disease Perception
Guide

Purpose

The Disease Perception tool helps you to understand people's perceptions of the disease you are interested in. Normally, this would be diarrheal diseases, but sometimes you might be trying to prevent outbreaks of something more specific, such as cholera. In particular, this tool helps you explore whether people perceive themselves to be at risk of disease (vulnerability) and how severe the consequences of getting disease are thought to be. You will also gain insights into how their perceptions of risk may have changed because of the crisis. This information will be useful when designing your intervention, as it is possible your priorities (handwashing to prevent diarrhea) are not the same as the community's priorities.

Requirements

Time

20–30 minutes per focus group discussion

Format

A minimum of three focus group discussions (FGDs). Consider doing more FGDs if you find your results from these groups are very different.

Participants

As a minimum, form one focus group with seven women and one with seven men.

Tip: Try to make sure the people in each FGD are similar. Mixing men and women, or young people and older people can affect power dynamics and make some participants reluctant to speak.

Materials

- Disease Perception cards (separate Word document)
- Disease Perception worksheet (separate Word document)
- Disease Perception consent script (at the end of the guide)
- Disease Perception decision making table (separate Excel sheet)

Roles

- *Facilitator:* one person to facilitate the activity and discussion
- *Scribe:* one person to capture key information from the participants, using the worksheet
- *Analysis team:* Several team members to analyze and discuss observations

Data Collection

Preparation

- Print out the Disease Perception colour cards. If you don't have a colour printer, use coloured pencils or pens to create cards similar to those provided. In the white square on each of the cards, write the meaning of the cards in the local language.
- Read the separate *Tips for Effective Focus Group Facilitation* guide in the *Quick Tips* section of the website.
- Rehearse the activity with others on your team.

Consent

Ask the participants for their consent:

- Introduce yourself.
- Explain what you are doing and why.
- Briefly describe the activity.
- Tell the participants how you will use their information.
- Assure them of confidentiality.
- Ask participants if they are willing to participate.
- Explain there will be no consequences if they do not wish to participate.

  (See a sample consent request script at the end of this guide.)

Activity

1. Decide what disease to focus on. If you are trying to reduce diarrheal diseases in general, use the tool as is. If you are working in a cholera or Ebola outbreak then replace each mention of diarrhea with the disease you are interested in.
2. Locate an appropriate space to conduct the FGD. Try to find somewhere that is private, conveniently located, and neutral. For example, asking risk-related questions in a room in a health centre might bias the way people answer, or holding an FGD at the village leader's house might make some people nervous to participate.
3. Introduce yourself and help participants to feel comfortable to talk to each other. To do this, you can get people to introduce themselves and say their favourite food, colour, or a particular skill they have. Use neutral questions versus more personal topics, as people may have recently been through difficult experiences.
4. Outline the ground rules for the FGD to participants, explaining there are no right or wrong answers. It is important for everyone to remain respectful of all opinions and not interrupt or talk over one another. To encourage everyone to share freely, request that they do not share what was discussed with anyone outside of the group after the session.
5. Ask the group to tell you the five illnesses they are most worried about. If diarrhea is not mentioned, ask if it is a concern and how much of a concern it is in relation to the other illnesses. Get them to rank the illnesses in order of which ones they worry most about to which ones they worry least about.
6. Explain that you would like to learn more about diarrhea in this community. Ask them to describe the symptoms of diarrhea and its causes (do not tell them what you know).
7. Summarize what the participants say so everyone is clear about what the word diarrhea will mean as you go through the activity.

8 Explain that you are going to do a guessing activity with them where they will each be asked to guess the chances of a bad thing happening.

9 For each of the following two questions, ask participants to point to the answer that best reflects their opinion:

Q1 "Do you think that someone in your family could get diarrhea in the next six months?"

Tips:

1 Get each person to point to the card that represents their guess. Where guesses differ, get people to explain their opinion.

2 Make sure everyone has a chance to speak by directly asking some of the quieter people for their opinion.

I think it will happen	I think it might happen	I don't think it will happen

Q2 "If someone in your family got diarrhea, do you think it could result in serious illness or death?"

I think it will happen	I think it might happen	I don't think it will happen

10 Tell the participants the scale changes a little for this question as follows:

Q3 "If you compare your family with other families that live near you, who is more likely to get diarrhea in the next six months?"

Other families	We are all at the same risk	My family

11 If you are working in an area where people have been displaced, ask the following question. If you are not, you can skip over this question. Explain that the scale changes again as follows for this question:

Q4 "All of you have been displaced recently. I want you to think of the place where you used to live and the place where you live now. Do you think your family is more at risk of getting diarrhea here or where you were before?"

We are at less risk here	Our risk is the same	We are at more risk here

12 Ask the following open-ended questions. No scale is needed.

Q5 "Imagine that a child in your family got diarrhea. What impact would this have on your day-to-day life?"

For Q5, ask additional probing questions such as:

- What affect might it have on your social life?
- What affect might it have on your productivity or income?

Q6 "What can families like yours do to prevent diarrhea?"

13 For the next question, explain that the scale changes again as follows:

Q7 "Since the crisis (for example, conflict, displacement, outbreak, or disaster), how often do people in your family wash their hands compared to before the crisis?"

If they are washing more often or less often, ask people to explain why their behaviour has changed.

Less often	An equal amount	More often

14 Thank the participants for their time.

Scribe:

1 As the activity progresses, complete the worksheet by circling the answers that most people agree with.

2 Take notes on the key points of the discussion. Pay special attention to any surprising comments or opinions.

Analysis

1 Discuss with your colleagues any surprising opinions that emerged from the discussion.

2 When you return to the office, open the project you have created in the Program Designer. Follow the instructions in the Program Designer to answer each of the questions about each of your FGDs.

3 The Wash'Em Program Designer will highlight key patterns emerging from your data. Read the section analysis to learn how to interpret these results and which behaviour change challenges to focus on.

Recommendations

1 After entering the results from all the tools you've used, click on the Generate Recommendations button in the Wash'Em Program Designer.

2 As a team, discuss the recommendations and how you plan to implement them.

Tool Limitations

Rating can be surprisingly hard to understand, particularly if participants have had limited formal education. If participants are struggling, do additional practice questions first.

In some cultures, a numerical explanation might make more sense. You can explain that *very unlikely* is roughly equivalent to there being a 0% chance of something happening, *unlikely* is the equivalent of it being about 50% likely, and *likely* is equivalent to it being 100% likely. They should choose the percentage they think the answer is closest to.

Participants will know that their answers may affect whether or not a program is delivered in their area. This belief may result in people over or underreporting their perceived risk. The best way to get around this problem is to listen carefully to the debates and discussions between participants.

Disease Perception Consent Script

It is important your participants are provided with appropriate details about why you are collecting information from them, what will be required of them, and how the information will be used. When using the Disease Perception tool, use the following explanation:

> Hi, my name is________and I work for________organization. We are visiting your community/camp to learn more about people's lives and behaviours. If you are willing to help us, we would like you to be part of a group discussion where we will ask you and five others from your community about the health issues affecting people around here. During the discussion, we will take notes. People in our organization will use the information you share with us. It will not be shared more broadly. We are not here to judge you, but just to learn from you. We will use the things we learn to design programs that will help people in communities/camps like yours. There are no consequences to you or your family if you choose not to participate. Do you wish to participate?

20.02.01

This guide was made possible by the generous support of the American people through the United States Agency for International Development (USAID).
The contents are the responsibility of LSHTM, ACF and CAWST and do not necessarily reflect the views of USAID or the United States Government
