# Supplementary material for: Using Wash’Em to Design Handwashing Programmes for Crisis-Affected Populations in Zimbabwe: A Process Evaluation
Source: Int J Environ Res Public Health. 2024 Feb 23;21(3):260. doi: 10.3390/ijerph21030260 (PMC10970461; doi:10.3390/ijerph21030260)
Supplement: Supplementary file 1 [file ijerph-21-00260-s001.zip › S4. Document_Rapid Assessment tool guide_Personal Histories.pdf]

Personal Histories
Guide

Purpose

This tool is used to generate a broad understanding about the experiences of populations affected by crisis. Often, a crisis can have an impact on people's sense of identity, the roles they play in their families and communities, their social relationships, their sense of control, and their ambitions. Remember, the first principle when providing humanitarian aid is: "Do no harm." Listening to people's stories helps you to apply this principle.

Requirements

Time
25–45 minutes per individual

Format
6 – 10 individual interviews

Participants
A minimum of six participants: try to select a diverse group of people. Try to get a mix of ages, gender, abilities, ethnicities, and religions. Also select participants based on geography, proximity to water sources, and access to resources. If you are working where there is an outbreak, the people you select should all have been personally affected by it. For example, a person who has recently recovered from cholera or a family member of the cholera case. For more guidance on choosing participants, read our *Selecting Participants* guide in the *Quick Tips* section of the website.

Materials
- Personal Histories worksheet (separate Word document)
- Personal Histories consent script (at the end of this guide)
- Personal Histories decision making table (separate Excel sheet)

Roles
- *Facilitator:* One person to facilitate the activity and discussion
- *Scribe:* One person to capture the participants' responses
- *Analysis Team:* Several team members to analyze and discuss the responses

Procedure

Preparation
- Select the worksheet (at the end of this guide) that is most appropriate in your context. Worksheet 1 is for use in displacement settings. Worksheet 2 is for use in disease outbreaks. Worksheet 3 is for use in disasters, and Worksheet 4 is for disease prevention.
- Print off one worksheet per participant and one additional worksheet for the interviewer. You may want to have some extras on hand.

- Read the interview tips below.
- Discuss how you would respond to participants if they become emotional or distressed and consider how you would handle any emotions you might feel.
- If possible, identify a safe and private space at the location you will be working, where your team can discuss the participant responses immediately after each interview (for example, your organization's vehicle).
- Rehearse the activity with your scribe.

Consent

Ask the participants for their consent:

- Introduce yourself.
- Explain what you are doing and why.
- Briefly describe the activity.
- Tell them how you will use their information.
- Assure them of confidentiality.
- Explain that there will be no consequences if they do not wish to participate.

See the a sample consent request script at the end of this guide.

Activity

1. Create a safe and comfortable space for the participants. Start with small talk and ask some neutral questions (for example, talk about the weather).
2. Explain your roles as interviewer and scribe.
3. Explain you want them to talk about the three different time periods indicated on the worksheet. Start by explaining what each time period means.
4. Ask the participant to draw a picture of themselves in each box on the worksheet to show how they looked and felt different during each of the time periods. Some of your participants may be illiterate or embarrassed by their drawing abilities. Remember, people who are illiterate are still able to draw. Assure them you are not concerned about the quality of the drawing and that you will not judge them. The rationale for having the participants draw a picture is to help them feel comfortable enough to open up about their experiences. Encourage participants to talk through what they are drawing and why. While it can seem daunting at first, most participants find it fun once they start.
5. Once the drawings are complete, begin by asking the participants to focus on the first time period. Move horizontally across the page, asking each question about each time period to understand the changes that have happened in each participant's life.
6. Before moving on to the next question, make sure your scribe has had time to write down the answers. Prompt the scribe if anything important has been left out.
7. Thank the participants for their time.

Scribe:

Take notes as each participant is speaking. You will not be able to write down everything someone says. Just try to capture the most important information.

Interview Tips

The following are some techniques for helping to put your participant at ease and getting the most from your interview.

- **Restate what you heard.** Before you go to the next section, briefly restate what you heard. Doing so shows that you listened to the person's story and allows the participant to correct any misunderstandings.
- **Use informal prompts.** Informal prompts are the questions, noises, and interjections that people make in a conversation, such as nodding, "uh-huh," or "wow!"
- **Make space.** Your job is to listen, so let your participants speak. If they go off topic, let them finish before redirecting them. If you try to control the conversation too much, you may miss an interesting story.
- **Speak softly** to convey that you understand the sensitivity and seriousness of their experiences.
- **Be conscious** that you look engaged in the conversation (in culturally appropriate ways). For example, don't yawn.
- **Discourage interruptions** from other people. If others come in, explain that you are having a private chat with this person right now, but you would be happy to speak with them later.

Analysis

1. Move to a private space where you and your scribe can recap what you heard.
2. With your partner, discuss the notes and your impressions immediately after the interview. Not delaying will allow you to remember what was said while it is still fresh in your mind.
3. Read horizontally across each participant's form. Make notes of the positive and negative shifts in participants' lives, roles, identities, social relationships, sense of control, and hygiene practices.
4. When you return to the office, open the project you have created in the Wash'Em Program Designer. Follow the instructions in the Wash'Em Program Designer to answer each of the questions about each of your interviews. In the Wash'Em Program Designer you will not find an answer that perfectly matches what the participant says. Your job is to identify the patterns of change that are most similar to what each person described.

Recommendations

1. After entering the results from all the tools you've used, click on the Generate Recommendations button in the Wash'Em Program Designer.
2. As a team, discuss the recommendations and make a plan to implement them. Take time to think about how you could adjust the recommendations to minimize harm or be more sensitive to the experiences your community has gone through.

Tool Limitations

The Personal Histories tool is a valuable way to learn more about context. However, you should be aware that participants may tell you about upsetting experiences. Talking about such things may be difficult for the participant, and hearing about them may also be difficult for your data collection team. Before you use this tool you should think about the following:

- Are there psychosocial support services that you could refer the participant to if needed?
- Are there certain verbal or body language cues that might indicate you should stop the activity? (For example, what will the team do if the participant starts crying? What are the implications of different courses of action?)
- What processes can you put in place to ensure that you support each other as a team emotionally? (For example, a daily debriefing where experiences are discussed or organizational psychosocial support services.)

Personal Histories Consent Script

It is important your participants are provided with appropriate details about why you are collecting information from them, what will be required of them, and how the information will be used. When using the Personal History tool use the following explanation:

> Hi my name is________and I work for________organization. We are visiting your community/camp to learn more about people's lives and behaviours here. If you are willing to help us, then we would like talk to you about how this emergency (for example, displacement/cholera outbreak/disaster) has affected your life. We will ask you to do a drawing to start, and then we will ask you some questions about your experiences. We know people around here have been through a lot of difficult experiences. If you think it will be too difficult for you to discuss what you have been through or if you do not feel comfortable sharing this type of information with us, then you should choose not to participate. The information you tell us will be used by people in our organization and will not be shared more broadly. We are not here to judge you, just to learn from you. The things we learn will be used to design programs that will help people in communities/camps like yours. There are no consequences to you or your family if you choose not to participate. Do you wish to participate?

20.02.01

USAID FROM THE AMERICAN PEOPLE · ACTION CONTRE LA FAIM · LONDON SCHOOL of HYGIENE &TROPICAL MEDICINE · CAWST

This guide was made possible by the generous support of the American people through the United States Agency for International Development (USAID). The contents are the responsibility of LSHTM, ACF and CAWST and do not necessarily reflect the views of USAID or the United States Government

Is the Personal Histories tool appropriate for my context?

Benefits of the Personal Histories tool:

1. As humanitarians, we are normally focused on relieving the suffering of many. When responding to the urgent needs of crisis-affected populations, we often do not take time to understand how the crisis has really affected people's lives. For example, when there is a cholera outbreak, we look at the number of cases, but we rarely take time to sit down with people who have had cholera and learn what it was like for them. The Personal Histories tool encourages you to make sure these personal experiences are at the heart of your program.
2. As humanitarians, we care about promoting handwashing behaviour because we know the public health impact it can have. But handwashing will never be seen as a major problem by crisis-affected populations as they have many other bigger worries. The Personal Histories tool will highlight to you these other concerns of the population. Understanding these concerns can be useful because handwashing does not happen in a bubble; all of these other worries could potentially impair people's ability to regularly wash their hands. Understanding these other worries might lead you to link your hygiene program with mental health programs or livelihoods programs in your area and this might make your program more acceptable and effective.

Examples

The Personal Histories tool was used during a cholera outbreak in Zimbabwe. Staff who had responded to several cholera outbreaks in the past, reported this was the first time they had ever sat down with a person who had had cholera and listened to their story. Among other things, the tool highlighted that getting cholera had a substantial impact on people's social lives. Some of the people who were interviewed using the Personal Histories tool reported that getting cholera had been a wake-up call for them and had prompted them to act more responsibly and prioritize things that mattered in life.

The tool was also used in northern Ethiopia in a camp for Eritrean refugees. Staff using the tools had been working in the camp for more than three years, but had never asked any members of the population about their experiences of displacement or what life was like in Eritrea. Staff were surprised to learn that even though life in the camp was not easy, the refugees were very happy to be there, they felt free, and they were hopeful for their future.

The Personal Histories tool was also used in the Democratic Republic of the Congo among internally displaced people. Here, staff found that hunger was a major barrier to handwashing. All daily activities were focused on just earning enough money to buy one meal a day for family members. Because people were so hungry, they were not able to remember to wash their hands before eating.

If any of the following conditions are TRUE for your context, you should NOT use the Personal Histories tool:

- There are no mental health or psychological services available for the population you are working with.
- The majority of the population (more than 60%) have been separated from friends or family within the past six months.
- The majority of your target population (more than 60%) knows someone who has died due to conflict, disease outbreaks, or disasters in the past six months.
- The majority of the population (more than 60%) have been exposed to violence within the past six months.

It is worth remembering that if you decide not to use this tool, you are still working with vulnerable people. Not using the Personal Histories tool could lead you to overlook important needs or create a program that could have unintended consequences.

If you are planning to use the Personal Histories tool, make sure to do the following:

- Involve people with mental health training in the data collection process if possible. These staff will be able to provide follow-up support to participants, if needed, and discussions with them will help contextualize the stories you hear.
- Identify mental health support services that you can refer your target population to.
- Be willing to engage with other humanitarian sectors to address issues that might emerge. For example, through using this tool in Iraq, we learned there were no special adaptations to hygiene kits for households where there was a person with disabilities. We were able to raise this issue with the organization responsible for distributions and the situation improved the following month.
- Develop a distress plan (see the training materials for more information).
- Clearly explain the method to each potential participant during the consent process.
- Remember that participants have the right to decline if they are not comfortable with the process you describe.
- After conducting your first Personal Histories interview, have another discussion with your team and reflect on whether you still think the tool is appropriate for use in your context. Stop if you are concerned.
