# Supplementary material for: Using Wash’Em to Design Handwashing Programmes for Crisis-Affected Populations in Zimbabwe: A Process Evaluation
Source: Int J Environ Res Public Health. 2024 Feb 23;21(3):260. doi: 10.3390/ijerph21030260 (PMC10970461; doi:10.3390/ijerph21030260)
Supplement: Supplementary file 1 [file ijerph-21-00260-s001.zip › S5. Document_Rapid Assessment tool guide_Touchpoints.pdf]

WashEm

Touchpoints
Guide

Purpose

Touchpoints are all of the ways you can touch the lives of the population you are working with. Understanding touchpoints is important because you can use existing social structures and ways of sharing information as some of the delivery channels for your intervention. This tool will help you identify the types of touchpoints that are present in your context. You can then prioritize which ones are likely to be the most effective in reaching your population.

Requirements

Time

20–40 minutes

Format

At least three focus group discussions (FGDs). Consider doing more FGDs if you find your results from both groups are very different.

Participants

Conduct at least one FGD with seven women and one with seven men. You may want to do additional FGDs with sub-groups of the population that you are particularly interested in (for example, with young people). For more information about selecting participants, read our *Selecting Participants* guide.

Tips:

- Try to make sure the people in each FGD are similar. Mixing men and women, or young people and older people can affect power dynamics and make some participants reluctant to speak.
- We recommend including people with disabilities in your focus groups as they may have different viewpoints to others in the community. If you do this, think about how you may need to adapt the process to ensure people with disabilities can participate equally.

Materials

- Touchpoints worksheet (at the end of the guide)
- Touchpoints population cards (at the end of the guide)
- Touchpoints image cards (at the end of the guide)
- Touchpoints consent script (at the end of the guide)
- Flip chart paper or a board (optional)

Roles

- *Facilitator:* one person to facilitate the activity and discussion
- *Scribe:* one person to capture the participants' responses
- *Analysis Team:* Several team members to analyze and discuss the findings

Data Collection

Preparation

- Print off one worksheet per focus group and one additional worksheet for the interviewer. Also print one copy of each of the category cards.
- Read the separate *Tips for Effective Focus Group Facilitation* guide in the *Quick Tips* section of the website.
- Rehearse the activity with your scribe.

Consent

Facilitator:

Ask the participants for their consent:

- Introduce yourself.
- Explain what you are doing and why.
- Briefly describe the activity.
- Tell them how you will use the information.
- Assure them of confidentiality.
- Explain there will be no consequences if they do not participate.

See a sample Touchpoints consent request script at the end of this guide.

Activity

1. Locate an appropriate space to conduct the FGD. Try to find somewhere that is private and conveniently located for the participants.
2. If possible, set out the cards in advance of the FGD starting. You should set these out based on the table in the worksheet: The population cards should go horizontally at the top and the touchpoint image cards should go vertically down the side. The simplest way of doing this is to lay the cards on the table or ground. However, you could also stick them on a flip chart or a board as you can see in Figure 1.

Figure 1
The WASH Cluster in the Philippines displayed the cards on a board for all the participants to see.

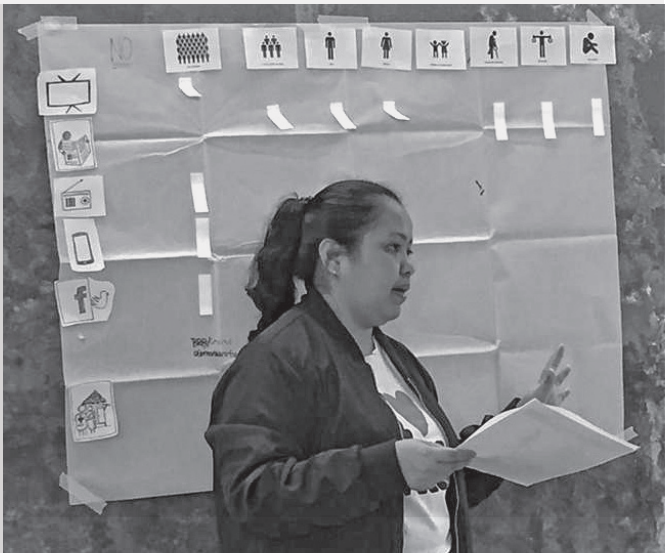

3 Introduce yourself and invite participants to do the same. One way is to ask them to introduce themselves, and say their favourite food, their favourite colour, or a particular skill they have. Use neutral questions instead of more personal topics, as people may have recently been through difficult experiences.

4 Explain how the FGD will work. Explain to participants there are no right or wrong answers to anything they discuss. Ask them to be respectful of all participants and not to interrupt or talk over one another. To encourage everyone to share freely, ask that they do not share what was discussed with anyone outside of the group after the session.

5 Explain you would like to learn about the services in their community, the ways people receive information and the places people spend their time. Explain that you want to learn about this so you can deliver programs that are suitable to their situation.

6 Introduce them to the cards. Take time to explain what the population cards are along the top of the table. Explain what each picture represents by reading out the explanation below the image. You do not need to explain each of the touchpoint cards at this stage.

7 Begin the activity by asking the questions in the left-hand column of the worksheet. If people say that a touchpoint (for example, television) is not present in their community, you can move on to the next touchpoint. If they say, "Yes, it is present," then use the population cards to get more information about the touchpoint. For example, if the participants say people have televisions, ask if many people have a television or a small number do. Then ask if men, women, children and people with disabilities get information from the television. Note that in the column on the far right. There are additional questions to answer if the first response was yes.

8 When asking the questions, make sure you give all the participants a chance to answer. Sometimes participants will disagree about the ways people get information in their community. If there are disagreements, summarize the different opinions then try to get the participants to reach an agreement.

9 The last section is designed to understand who in the community people admire, as these people are likely to influence the behaviour of others. To understand this, ask "Who do people in this community respect or admire?" Encourage people not to name specific individuals, but figures. You can mention the following examples if this helps: parents, friends, work colleagues, siblings, community leaders, religious leaders or neighbours.

Scribe

1 The scribe should place a number in the boxes that correspond to the participants' answers. If the participants say 'no' to one of the questions, only the first box should be marked with a zero. If participants say 'yes' the scribe should write down the corresponding number in as many boxes as are applicable (based on the participants' description of how many people use that touchpoint).

2 The scribe should also note the responses to the additional questions in the right-hand column.

3 For the last question about respected people, the scribe should write down the two most commonly mentioned figures.

4 Thank the participants for their time.

Analysis

1 Begin by asking if there was anything surprising, or that particularly stood out.

2 Looking at the worksheet, identify which touchpoints are able to reach the majority of people. Do this by considering which touchpoints reach 'lots of people' as well as which touchpoints were able to reach all of the subgroups of people. Sum the total number of points awarded in the total column (this should not exceed nine points). The higher the total score, the more effective the touchpoint will be. Use this information to enter the data into the Wash'Em Program Designer. If you have more than three touchpoints with the same highest score you will need to use your judgement to select the three touchpoints that seem to be the most effective for your target population.

3 If you encounter a Section Conflict and are unable to carry out more FGDs, to help you resolve the conflict, go back to your completed worksheets and look to see which of the options available in Q46 feature highest in the ranking carried out in the FGDs. For example, if Health workers was placed higher than School, Community meetings and Women's groups, select this option.

Recommendations

1 Reflect on the touchpoints that seem to reach the most people. Do they have any limitations? Are there other factors that could make it difficult to use them in your program? For example:

- If you were to select the three touchpoints that reach the most people, would you leave out any of the sub-groups, such as women, children or people with disabilities?
- Does your donor, or the government, require you to deliver your program in a certain way?
- Are there safety or security reasons for not using certain touchpoints? For example, in a disease outbreak, you should not use large community events, because they could increase the spread of the disease.
- Which touchpoints would work best with your behaviour change challenge activities?

2 Based on your discussions, select the three most effective, or most feasible touchpoints
and enter these in the Wash'Em Program Designer.

3 After entering the results from all the tools you've used, click on the Generate Recommendations button in the Wash'Em Program Designer.

4 As a team, discuss the recommendations and make a plan to implement them.

Touchpoints Consent Script

It is important your participants are provided with appropriate details about why you are collecting information from them, what will be required of them, and how the information will be used. When using the Touchpoints tool, use the following explanation:

> Hi my name is________and I work for_________organization. We are visiting your community/camp to learn more about people's lives and behaviours here. If you are willing to help us, we would like you to be part of a group discussion where we will ask you and five others from your community about the services available in your community, how you get your information, and how and where people socialize. During the discussion, we will take notes. The information you tell us will be used by people in our organization and will not be shared more broadly. We are not here to judge you, but just to learn from you. The things we learn will be used to design programs that will help people in communities/camps like yours. There are no consequences to you or your family if you do not wish to participate. Do you wish to participate?

This guide was made possible by the generous support of the American people through the United States Agency for International Development (USAID).
The contents are the responsibility of LSHTM, ACF and CAWST and do not necessarily reflect the views of USAID or the United States Government
