# Supplementary material for: Using Wash’Em to Design Handwashing Programmes for Crisis-Affected Populations in Zimbabwe: A Process Evaluation
Source: Int J Environ Res Public Health. 2024 Feb 23;21(3):260. doi: 10.3390/ijerph21030260 (PMC10970461; doi:10.3390/ijerph21030260)
Supplement: Supplementary file 1 [file ijerph-21-00260-s001.zip › S6. Document_In depth Interview guide for implementing staff.pdf]

Wash'Em

In depth Interview guide

Understanding experiences of using the Wash'Em process to design hygiene programmes in crises.

Overview

This document outlines interview guides for 3 rounds of interviews with staff involved in Wash'Em training, data collection and activity implementation. These rounds of interviews will fall in advance of the training, following the data collection and at the end of the activity implementation.

These guides are designed to be used flexibly by interviewers and questions will not be asked in a structured or chronological manner. Questions may be skipped or asked in a different order as the interviewer will be guided by participant responses and will probe accordingly. Questions may also be skipped if it is known that a particular staff member was not involved in part of the process because of their stated role. Additional questions and themes may be added to probe on particular points that the participant raised in previous interviews or in relation to specific parts of the implementation process that were not forecasted at the beginning of the process.

The guides are structured according to the phases of Wash'Em uptake and use and therefore follows a chronological process of the use journey.

Participants should be given a brief overview of the structure of the interview at the beginning.

Questions in italics are designed primarily as additional probes but are unlikely to be systematically asked to all participants.

Interview 1: Prior to Wash'Em training

Primary focus areas: Prior experiences of participants, information on the crisis context and broader programming, planning for Wash'Em and awareness and expectations of the Wash'Em process.

Prior experiences

1. Can you start by telling me a bit about your background including your current role and responsibilities and your prior experiences related to humanitarian hygiene programmes?
   - *Did you play an active role in hygiene programme design or modifying programmes? Why or why not?*

2. Thinking about your experiences up until now, can you reflect on the strengths and weaknesses of the hygiene programming that your organisation has implemented or the strengths and weaknesses of hygiene programmes that you have observed other organisations doing?
   - *Thinking of your prior hygiene programming experience, which part of the programme design or implementation process was most challenging?*
   - *Thinking of your prior hygiene programming experience, do you feel these programmes were seen as relevant and appropriate by populations and in your view did they work to change behaviour? Why or Why not?*

Crisis context

3. Can you tell me about the humanitarian situation where you are working and where you will be implementing Wash'Em?
4. What challenges or opportunities did you foresee for promoting hygiene in this area?

Awareness about Wash'Em and expectations

5. Can you tell me what you know about Wash'Em and where you first learned about the process?
6. Why did your organisation decide to use Wash'Em?
7. In what ways do you think Wash'Em might be different to previous processes you have used for hygiene programme design?
   - *Do you have any concerns about using Wash'Em or expectations about which part of the process might be challenging?*
   - *Which parts of the Wash'Em process are you excited about and why?*
   - *Do you have any expectations about how easy Wash'Em will be to use or how much time and capacity may be needed from staff?*
   - *Do you think Wash'Em is likely to result in hygiene programmes that are more acceptable to crisis-affected populations or which are more likely to result in behaviour change?*
8. How do you envisage Wash'Em being a part of the broader project you are running?
   - *How will you integrate Wash'Em into broader project commitments and timelines?*
   - *How will you design the other behaviour change components that are not handwashing related?*
9. Have you had to make specific plans to incorporate Wash'Em into your broader project plans?
   - *What adjustments had to be made in terms of proposal writing, timelines, budgets, staffing and monitoring.*

Wash'Em

Interview 2: After training, data collection and data analysis

Primary focus areas: Experiences of the training, data collection process, data summary and recommendations generated; and plans and expectations for adapting and implementing the activities;

Understanding of the process:

1. How has your view or expectations of Wash'Em changed following the training and use of the tools?
   - *How do you feel the Wash'Em process differs from previous approaches you used for hygiene programme design?*
   - *What do you think about the amount of time that it takes to conduct the training and use of the tools?*
   - *What did you feel was most appealing or interesting about the Wash'Em process?*
   - *Are there any aspects of Wash'Em that you are sceptical about, if so what?*
   - *In your own words, what do you understand to be the main purpose of using Wash'Em*

Training on the Rapid Assessment Tools:

2. How did you find the Wash'Em training?
   - *What were the main skills that you feel you developed during the training?*
   - *Do you feel the training changed your opinion of behaviour change programming? In what way?*
   - *Did you feel that the training involved the right number of people and mix of skills to carry out the Wash'Em process?*
   - *Did you feel that any of the training components were too long or too short?*
   - *What did you think about the training materials used? Would other formats have aided your learning?*
   - *How effective was the trainer in guiding you through the training?*
   - *If you were to do the training again in the future, what would you add or do differently? Are there aspects of the training that could be made easier to understand?*

Using the Rapid Assessment tools

3. Can you describe your experiences of using the Wash'Em Rapid Assessment Tools and how they compared to other needs or behavioural assessments you may have used in the past?
   - *Were some of the tools easier and more rapid than other rapid assessment tools you have used previously?*
   - *Which of the tools did you feel generated the most useful insights about behaviour?*
   - *Can you share one insight that was generated that you found particularly interesting or surprising?*
   - *What parts of the tools didn't you like?*
   - *What aspects of the tools could be improved in your view?*

Summarising data in the decision making tables and using this to answer questions in the software

4. How did you go about the process of analysing the data that came from the Wash'Em Rapid Assessment Tools?
   - *What was your role in summarising and analysing the data?*

- *Was it easy to understand how to do the analysis using the decision making tables?*
- *What were the main questions that arose during the analysis?*
- *Wash'Em requires you to identify common patterns in behaviour and perceptions across your participants - did you encounter any challenges in identifying and interpreting these patterns?*

5. What was your experience like with entering your summary data into the Wash'Em software?
   - *Did you find the software easy to navigate?*
   - *Did the software function as you expected? Were there functions that you think are missing?*
   - *Did you find it hard to answer some of the questions in the software?*
   - *Did you understand why all of the questions in the software were being asked?*
   - *Based on your understanding how does the Wash'Em software make programmatic recommendations based on the responses you give?*

Interpreting recommendations and translating them into programme implementation

6. As you read through the recommendations in the software, what were your first impressions?
   - *Were the recommendations easy to understand?*
   - *Did you feel the recommendations addressed the behavioural challenges identified in the Rapid Assessment tools?*
   - *In what ways were the recommended activities different from activities within your existing programmes or the types of hygiene programming that you are used to seeing in your region?*
   - *Did you think some of the recommendations were not appropriate given restrictions associated with the COVID-19 pandemic? Why?*
   - *Could anything be done to improve the way recommendations are presented and described within the software?*

7. After reading the recommendations, what steps did you take to plan for how the activities would be incorporated into your project?
   - *Did you have to adapt activities in any way? Why was this necessary and how were adaptations made?*
   - *Did you have to plan for the development of materials or the procurement of products? What did this process entail and what was your role?*
   - *Who did you have to get approval from to utilise recommendations within your programme (e.g. senior managers or donors)?*
   - *Did you have to make any adjustments to your staffing, budgeting or timelines to facilitate implementation of the recommendations?*
   - *What could be done to make it easier to transition between having these recommendations and actually implementing them?*
   - *How did you try to integrate the Wash'Em activities into broader WASH or COVID-19 prevention programmes/ multi-sectoral programmes/ or existing community or delivery mechanisms?*

Expectations related to project implementation:

8. Do you think Wash'Em is likely to result in hygiene programmes that are more acceptable to crisis-affected populations or which are more likely to result in behaviour change?

- *Based on your experiences so far, do you think you would be likely to use the Wash'Em process again in the future?*

9. What do you see as the main challenges in implementing Wash'Em activities within your project?

Interview 3: After project implementation

Primary focus areas: Experiences implementing Wash'Em designed activities.

Strengths and limitations of Wash'Em designed activities:

1. Can you tell me what has been going well with implementing the Wash'Em designed activities within your project?
   - *Which activity in your view has been most effective? Why do you think this?*
   - *Which activity has been easiest for your teams to implement?*
   - *What feedback have you had from community members to indicate the acceptability of the Wash'Em designed activities?*
   - *As you have been implementing the activities have you noticed anything which indicates changes in behaviours and attitudes around handwashing?*
   - *Do you think the Wash'Em designed activities are likely to be more or less effective than previous hygiene promotion activities that you have tried?*
   - *Do you feel that you used a more diverse range of delivery channels compared to your prior hygiene promotion work?*

2. Can you give me one example of a challenge you have faced with implementing any of the Wash'Em designed activities?
   - *Which activity in your view has been least effective? Why do you think this?*
   - *Which activity has been hardest for your teams to implement? Why?*
   - *Have community members raised any concerns about activities?*
   - *Do you feel that any of the activities were unclear or left community members confused? Give examples if possible.*
   - *Have the community members mentioned any other barriers to handwashing behaviour that were not identified by the Wash'Em process?*

Time, logistics and capacity

3. How do the Wash'Em activities compare to prior hygiene promotion work/your expectations in terms of the amount of staff time they take to implement?
   - *Do you feel that you spent more time interacting with populations while implementing Wash'Em as compared to other hygiene promotion activities you may have tried in the past?*
4. How do the Wash'Em activities compare to prior hygiene promotion work/your expectations in terms of the logistics needed to support their implementation?
   - *Probe in terms of budget, planning, and procurement.*
5. *Do you feel that you and the rest of the team had the sufficient skills necessary to implement the Wash'Em activities?*
   - *What particular skills did you feel are needed for implementing Wash'Em designed activities?*
   - *Are there ways that these skills can be better supported in your view?*

Reflections:

6. What about the implementation of what Wash'Em process would you do differently if you were to try it again?

7. On reflection, what was the hardest stage of the Wash'Em process?
8. If you were encouraging another humanitarian to consider using Wash'Em, how would you describe it to them or what would be the primary selling point you would highlight to them?
