# Supplementary material for: Using Wash’Em to Design Handwashing Programmes for Crisis-Affected Populations in Zimbabwe: A Process Evaluation
Source: Int J Environ Res Public Health. 2024 Feb 23;21(3):260. doi: 10.3390/ijerph21030260 (PMC10970461; doi:10.3390/ijerph21030260)
Supplement: Supplementary file 1 [file ijerph-21-00260-s001.zip › S7. Document_Focus Group Discussion Guide.pdf]

Focus Group Discussion guide

Understanding the perceptions of crisis-affected populations in relation to a Wash'Em designed hygiene programme.

Overview

This document outlines an FGD guide to be used at the end of the programme implementation. This guide outlines the general process of the FGD and the topic areas to cover. However it should be used flexibly by research staff to allow for probing based on participant responses and discussion. Additional questions and themes may be added based on the final design of the Wash'Em programme.

The guide also outlines the process for selecting participants through a screening questionnaire. This may be modified based on the results of the Wash'Em Rapid Assessment tools in this context so that the questions can more closely measure exposure.

Screening

1. Potential participants should be approached individually, provided with an overview of the FGD and then they should be asked the screening questions to assess their exposure to the intervention. If the participant meets the eligibility criteria they should be then informed more thoroughly about the FGD (information form) and the consent process should be undertaken prior to them arriving at the FGD.

**Demographics**	
1. Gender	• Female<br>• Male<br>• Prefer not to say
2. Age	• 18-25<br>• 26-59<br>• 60+
3. Do you consider yourself to have a disability or a functional limitation?	• Yes<br>• No<br>• I don't know<br>• Prefer not to say
**Screening questions**	
4. In the last 3 months have you had a village health volunteer or staff member from ACF or Africa Ahead visit you or your community to talk	• Yes. If so Describe: _________________<br>• No<br>• I don't know

about handwashing?	
5. Have you recently seen any information on television or radio about handwashing ?	• Yes. If so Describe: ________________<br>• No<br>• I don't know
6. Have you recently attended a Community Health Club meeting where the topic was handwashing?	• Yes. If so Describe: ________________<br>• No<br>• I don't know
7. Have you participated in any activities involving XXXXX that are related to handwashing?	• Yes. If so Describe: ________________<br>• No<br>• I don't know
**Assessing Eligibility**	
Did the participant answer yes to 2 or more of the screening questions AND their descriptions aligned with actual implementation activities?	• Yes<br>• No
Allocation to FGD group:	• group of younger men (18-25 years)<br>• group of younger women (18-25 years)<br>• group of adult men (26-59 years)<br>• group of adult women (26-59 years)<br>• group of older men (60+ years) or adult men with disabilities (25+ years)<br>• group of older women (60+ years) or adult women with disabilities (25+ years)
Does the participant consent to be part of the FGD?	• Yes<br>• No

---

Introduction to the FGD session:

1. For the FGD there should be one research assistant who acts as the primary facilitator of the discussion and another research assistant who is the scribe. The scribe should capture the key things that everyone says.
2. As participants arrive, ask them to complete a sheet with their basic socio-demographic details on it (see end of this document).
3. Set up the space to mitigate COVID-19 transmission. This should include hand cleaning on entry and mask use by all participants and research staff. Chairs should be located at a distance from each other.
4. To start, get participants to introduce each other and say their favourite food, colour or a particular skill they have. These are all fairly neutral questions and are preferable over more personal topics given the experiences people may have recently been through.
5. Then explain the rules of the focus group these should include the following:

a. Explain the COVID-19 rules and why it's important these are adhered to throughout the session.
b. Explain that there are no right or wrong answers to anything we discuss today and it is important that we respect the opinions of others in the group. The facilitator should strongly encourage all participants to contain the opinions within the group attending the FGD.
c. In this discussion we are interested in hearing everyone's opinion. So we will encourage you all to take turns speaking and you should try not to talk over or interrupt other people while they are speaking.
d. We want you to feel comfortable sharing your opinion with us and so we would ask that all of you agree to not share what we discuss here with those outside the group after we finish.

Handwashing barriers

6. Start by asking the participants whether there are any factors that make it challenging for them and their families to wash their hands with soap. Even if they personally find it easy, get them to think about factors that might make it difficult for some other people in their communities.

7. What kinds of solutions can they think of to address these barriers? What kind of support would be needed to overcome the barriers identified?

8. Thinking back over the last decade do you recall many programmes in your area that have focused on trying to improve handwashing behaviour? What did these programmes typically involve? Which organisation were they delivered by? Who was targeted? What was the nature of activities? How long did these programmes run for? What did you like or dislike about these programmes?

Reflections on Intervention components

9. Start by describing to participants the various components of the Wash'Em designed hygiene programme. This should be done by showing visuals of each of the intervention components. As these are described, ask participants to describe whether they recall these components. Explain that you would like to discuss each component in turn. If they were exposed to the component it would be useful if they could draw on this experience. Ask participants what they liked and disliked about the intervention component that they were exposed to. If they were not exposed to these components they can still share their opinion based on the description provided.

10. Introduce the scale (see Image 1). Explain that the scale goes from 1-5 and that we are going to use to assess each component against different criteria. In each case 1 will represent the worst situation and 5 the best situation, with points 2-4 being points along this continuum. For each of the criteria that you ask about you would like them to reflect on the intervention activity, discuss their opinions of it and then make a group decision about how to grade it. If people disagree, that's ok, just explain why you feel differently and see if you can come to a consensus.

Image 1: *Scale - Print the scale below in colour and as large as possible before the FGD*

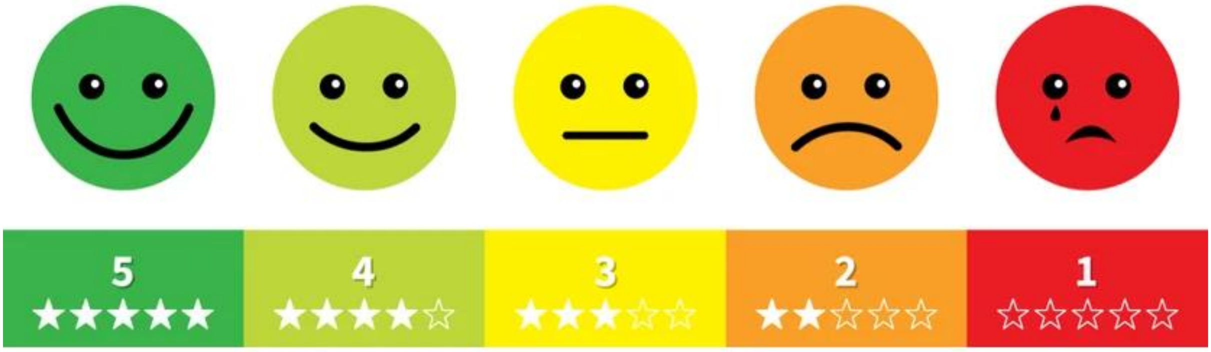

11. Ask about each of the following criteria for each intervention component:

- Is the message expressed within this component **easy to understand**? (range from very difficult to understand to easy to understand). In discussing this criteria make sure to ask participants what they think the message is.
- Was the component **easy to participate in**? (range from very difficult to participate in to easy and convenient to participate in). In discussing this criteria ask participants about potential barriers to participation such as a) literacy, b) the need to travel to a particular location c) the need to have access to mass media (e.g. radio)
- How likely are they to **mention it to a friend or family member**? (range from would not mention it to would definitely mention it). In discussing this criteria ask people what and how they are likely to mention it in conversation.
- How likely are you to **recall the message one month later** when they are busy with other concerns and priorities? (range from will not remember to will definitely remember). In discussing this criteria ask what makes it memorable.
- How likely is this component to **influence my handwashing behaviour**? (range from will not affect my behaviour to will definitely affect my behaviour). In discussing this criteria ask why they think it's likely to influence their behavior.
- How **relevant** does this component seem in relation to **your experiences, concerns and the barriers I face when handwashing**? (range from completely irrelevant to highly relevant). In exploring this criteria ask about what made it seem relevant.
- How well do you think **the implementing organisation understands the needs of this community** based on this component? (range from poor understanding to detailed understanding). In discussing this criteria explore what indicated this level of understanding.
- How **offensive** is this activity to people in this community? (range from 1 - highly offensive to 5 - not offensive). In discussing this criteria explore anything that was offensive about the component.

12. Ask participants for any ideas they have to improve each of the components.

13. Repeat steps 11 and 12 for each intervention component.

14. Thank participants for their time and see if they have any questions which they wish to ask.

Socio-demographic Information Sheet

Complete the sheet below as participants arrive at the session.

	Thinking of the last month, has your family always had enough water to meet their daily needs? (Y/N)	At home at the moment do you have soap available in your household? (Y/N)	How many people, including yourself, live in your household?	Does your household have access to a private latrine or shared toilet facility?	Do you, or any members of your household, struggle to wash their hands independently?
Participant 1	• Yes<br>• No	• Yes<br>• No		• Yes - private<br>• Yes - shared<br>• No	• Yes, Me<br>• Yes, other family member<br>• No
Participant 2	• Yes<br>• No	• Yes<br>• No		• Yes - private<br>• Yes - shared<br>• No	• Yes, Me<br>• Yes, other family member<br>• No
Participant 3	• Yes<br>• No	• Yes<br>• No		• Yes - private<br>• Yes - shared<br>• No	• Yes, Me<br>• Yes, other family member<br>• No
Participant 4	• Yes<br>• No	• Yes<br>• No		• Yes - private<br>• Yes - shared<br>• No	• Yes, Me<br>• Yes, other family member<br>• No
Participant 5	• Yes<br>• No	• Yes<br>• No		• Yes - private<br>• Yes - shared<br>• No	• Yes, Me<br>• Yes, other family member<br>• No
Participant 6	• Yes<br>• No	• Yes<br>• No		• Yes - private<br>• Yes - shared	• Yes, Me<br>• Yes, other family member

				● No	● No
Participant 7	● Yes<br>● No	● Yes<br>● No		● Yes - private<br>● Yes - shared<br>● No	● Yes, Me<br>● Yes, other family member<br>● No
