# Supplementary material for: Using Wash’Em to Design Handwashing Programmes for Crisis-Affected Populations in Zimbabwe: A Process Evaluation
Source: Int J Environ Res Public Health. 2024 Feb 23;21(3):260. doi: 10.3390/ijerph21030260 (PMC10970461; doi:10.3390/ijerph21030260)
Supplement: Supplementary file 1 [file ijerph-21-00260-s001.zip › S8. Document_Results of the Wash'Em Process - Activities recommended..pdf]

Activity name	Being pulled in all directions
Cost	Inexpensive
Difficulty	Moderately difficult
Delivery time	45 minutes
Short description	This is a participatory play about a hardworking mother. It can be delivered at a community event.
Objectives	• Help people realise that even in challenging circumstances or when you are busy, it is important to make time for handwashing<br>• Associate handwashing with feeling comfortable and refreshed
Procurement required	None
Creative work required	Yes
What you need	• Cards with drawings on them depicting different daily activities<br>• String<br>• Hole punch<br>• Laminator (optional)
How to do it	Step 1: Ahead of time, prepare a set of about 10 cards. Each card should depict a common daily activity in your area. This could include things like sweeping the house, collecting water, preparing food, getting children ready for school, doing laundry, washing the dishes, going to work, etc. Try to focus on tasks that are hard work but which a good parent would do on a regular basis. You may want to laminate each card if you have a laminator.<br><br>Step 2: Punch a hole in the top of each card and tie the end of a piece of string to it. Cut the string to about one meter (approximately 3 ft) in length. At the other end of the string, tie a loop that is large enough for a hand to fit through.<br><br>Step 3: Identify a staff member to act as the main character, or work with a local theatre group. Practice the play before going to the community.<br><br>Step 4: Organise a community event<br><br>Step 5: Ask for some volunteers from the crowd (choose one volunteer for each of the cards). Get them to line up and give them each a card which has a string attached.<br><br>Step 6: Get the main character to deliver the following story:<br>• 'I am a proud woman and everything I do, I do for my family because I love them and want what is best for them. But sometimes it's exhausting. I wake up early and sweep the house to make everything neat.'

	• The volunteer who has a card depicting a broom should walk up to the main character and place the loop of the string over her hand so that it is now attached to her arm. The volunteer should continue to hold the other side of the card while the main character acts out the rest of the play.<br>• 'Then I walk a long distance to collect water for my family. Carrying the water home is heavy and exhausting.' The volunteer with a card that has a jerry can on it comes forward and places it on the main character's arm. Again the volunteer holds onto the other end, pulling the main character in different directions.<br>• Continue to tell the story of a mother's day and all the chores that they complete. Keep adding cards to the mother's arms.<br>• At the end of recalling her day the mother should say: 'This is just a normal busy day for me. Sometimes I feel like I am being pulled in all directions.' Get each of the volunteers to pull her from side to side with the cards. The main character should move as if she is being pulled to attend to all her different priorities.<br>• 'When you are so busy it is hard to take time for yourself or to stay focused on what is important. When you are tired from working hard it is understandable to want to cut corners. But if you really care for your family there are some things that you cannot skip.'<br>• The volunteers should let go of the cards and the main character should slowly remove each card from her hands._ 'All of these daily chores I do with my hands. If I do these chores when my hands are dirty, smelly and unclean my family won't benefit and my house will remain a mess. One of the things I always make time for is handwashing with soap. It gives me that few moment of me time that allow me to continue working hard and caring for my family as best I can. No matter how busy you are, you can always make time for handwashing with soap.'_<br>• The main character should then wash her hands with soap. At the end she should smell her hands and sigh with happiness. 'That feels better'.
Closing content	
Tips	• Prepare well: This activity can easily become chaotic. If this happens, the population may not understand the story or see the point. To avoid this, make sure your team are well prepared. In addition to the main character, you may want to have someone who directs the volunteers. Alternatively, you could preselect some volunteers from the community or work with a local theatre group so that the volunteers know what to do in advance.<br>• Involve the community leaders: Before the community even you might want to meet with the community leaders and discuss what you plan to do and share some of the photos. This briefing can help ensure that the village leader is supportive and will encourage community members to take action. However before you involve these people, take time to understand from others in the community how they are perceived.<br>• Plan community meetings: Community meetings are normally a great way of reaching lots of people. However, in some situations such as a disease outbreak (e.g. cholera or Ebola), or when working in unsafe

	areas, they should be avoided or planned very carefully so that no one is put at risk by attending. Make sure to take time to assess whether large community meetings might put your populations at risk. Remember that you may have to do multiple community events if you want to reach everyone. You may also have to make special efforts to ensure everyone is invited (for example, people with disabilities and older people often get left out of community meetings because it is harder for them to travel to the meeting location).

Activity name	The power of soap
Cost	Inexpensive
Difficulty	Easy
Delivery time	20 minutes
Short description	Get people to rub glitter on the hands and then show that water alone isn't sufficient to remove it - only handwashing with soap will get all the glitter off.
Objectives	• Help people realise that soap is the magic ingredient that gets hands really clean: washing hands with water alone just isn't good enough.
Procurement required	Yes
Creative work required	None
What you need	• Glitter and petroleum jelly (or turmeric powder and cooking oil)<br>• Liquid soap
How to do it	Step 1: Bring several neighbouring households together.<br><br>Step 2: Ask for two volunteers. Get them both to rub a teaspoon of petroleum jelly/oil into their hands.<br><br>Step 3: Now add a teaspoon of fine glitter/turmeric powder to their hands and get them to rub it all over their hands. As they do this, their hands will turn red/yellow (turmeric) or they will be covered in glitter. Explain that now we are going to see what works best to get really clean hands. Ask the first volunteer to wash their hands with water only. Check their hands after 30 seconds and get them to show their hands to others in the audience. Water should have removed the powder but will not have removed the turmeric colour from their hands. Water alone will not have removed the glitter. Now ask the second volunteer to wash their hands with soap and water. Again get them to show the audience their hands afterwards. This time they should be completely clean. Explain to the audience that if you clean your hands with water only they may look clean but water alone will not get rid of all the germs.

	‘Soap is the extra special ingredient that gets hands truly clean’.
Tips	• Practice first: Try this activity out in your office first so that you learn how much glitter/tumeric you need and how much oil/petroleum jelly you need.<br>• Choosing products: For this activity you need to buy either glitter and petroleum jelly (vaseline), or tumeric and cooking oil. The activity will work less well if you use glitter with oil, or turmeric with petroleum jelly.

Activity name	Pledging
Cost	Inexpensive
Difficulty	Easy
Delivery time	15 minutes
Short description	Work with households to develop a pledge.
Objectives	• Make handwashing with soap a cultural norm<br>• Create neighbourhood support systems to encourage handwashing<br>• Associate handwashing with being a good parent or being respected and polite
Procurement required	None
Creative work required	Yes
What you need	• Poster<br>• Markers
Opening content	Publicly committing to practice a behaviour can make people much more likely to practice it.
How to do it	Step 1: Use the results from the motive activity to decide whether it is most appropriate in your context to link handwashing to being a good parent, or being polite and respected.<br><br>Step 2: Bring several neighbouring households together.<br><br>Step 3: Ask the participants how they show respect or how they show their children that they care. Try to get people to identify simple day-to-day actions. Examples for respect might include: the way I greet others or the way I welcome guests. Examples for being a good parent might include spending time playing with my child, or helping my child to learn.<br><br>Step 4: Ask whether handwashing with soap is also a way of showing respect: ‘Is it rude to shake hands with someone if your hands are dirty?’; ‘Does a good

	parent always wash their hands with soap?'; 'Does a good parent remind their children to do the same?'<br><br>Step 5: Explain that you are going to write down a list of the things that they suggested. This list can be displayed near their houses to remind them of the important actions they take every day to be more respectful/a better parent. Make sure handwashing with soap is included on the list.<br><br>Step 6: Ask if they agree that the things that you have written down are important. If so get them to make a pledge. An example of a pledge could be: 'I commit to always respecting others. I promise to show respect to others by greeting them warmly, listening to what they have to say, and always washing my hands with soap before cooking or eating, and after using the toilet.'<br><br>Step 7: Pledging can be enhanced by giving people a certificate once they have made the pledge.
Tips	Pledging works best when:<br>• It is culturally adapted. Take time to understand a culturally appropriate way of making a pledge (e.g. putting hands on hearts). One way to find out about this is by asking what people normally do when they make a promise or when they sing their national anthem.<br>• Handwashing is aspirational. Make sure that the pledge is not just about practicing handwashing but links to the broader inspirational idea in your guiding story.<br>• The behaviours are specifically stated. For example, don't just say: 'handwashing'. Say: 'Handwashing with soap before cooking or eating and after using the toilet.'

Activity name	Commitment card
Cost	Inexpensive
Difficulty	Moderately difficult
Delivery time	1 month
Short description	It can often be hard for communities with few resources to make dramatic changes. This activity focuses on breaking down the process of improving handwashing behaviour into small, doable actions.
Objectives	• Help people feel that are able to make meaningful improvements to their lives through small changes<br>• Help people to take ownership of building or improving their handwashing facilities
Procurement required	None

Creative work required	Yes
What you need	• Printed commitment cards<br>• Stick on stars<br>• Soap (optional)
How to do it	Step 1: Create a commitment card. Each card should have images depicting a series of simple, small, doable actions to improve handwashing. These might include some of the following actions:<br>• Nails are kept short and cleaned<br>• Remind others in my family to wash their hands<br>• Soap is kept near the toilet<br>• Soap is kept near the kitchen/eating place<br>• Water is kept near the toilet<br>• Water is kept near the kitchen<br>• Create handwashing reminders<br>• Build a handwashing facility<br>• Decorate the handwashing facility<br>• Adjust the handwashing facility so that it can be used by all family members<br>Make sure the commitment card looks nice so that people want to display it in their homes.<br><br>Step 2: Give each family a commitment card and work with them to display it somewhere outside their house where others can see.<br><br>Step 3: Discuss the things that they may be able to do straight away and which things they will tackle next. Develop a time plan for achieving each activity. Discuss what things they will need to do or buy or change to achieve each activity.<br><br>Step 4: Revisit households to assess their progress, recognise achievements and support them. Place stick-on stars next to each activity that is achieved. Create a competition between households to see who can manage to complete the most small, doable actions. You may also like to reward households who achieve multiple doable actions by providing them with additional soap.
Closing content	You can find out more about this approach [here](https://www.fhi360.org/resource/small-doable-actions-feasible-approach-behavior-change-learning-brief)
Tips	• Make the commitment visible: When you are explaining the commitment cards, make sure to bring several households together. This will create peer pressure to take action. Also make sure each family displays their commitment card in a location that is visible to other household members. This will help to create a sense of competition to achieve each action.<br><br>• Pay attention to barriers: This activity requires hygiene promoters to do multiple visits to households in order to support families to take action.

	During visits pay attention to common problems or factors which prevent families from taking action. You might find that these are issues you need to address through other aspects of your programming.<br><br>• Link this activity: This activity can work best when linked to other areas of your WASH programming. Consider what other _small, doable actions_ can be taken to improve toilets, improve water treatment and storage, disposal of waste, or generally make the home a cleaner, safer environment.

Activity name	Dye on food
Cost	Inexpensive
Difficulty	Moderately difficult
Delivery time	30 minutes
Short description	Using food dye, show how germs can easily spread from hands to food.
Objectives	• Help people realise that even a few germs on your hands can spread and contaminate the food that you and your family are eating.
Procurement required	Yes
Creative work required	None
What you need	• Typical local foods that are eaten or prepared with people's hands<br>• Coloured food dye<br>• Plates<br>• Handwashing facility and soap
Opening content	This activity works best in countries where people eat with their hands, using a staple foods like fufu, nsima, ugali, chapati or bread.
How to do it	Step 1: Organise a community event.<br><br>Step 2: Set up a table with some of the typical food product available (for example, fufu that has been prepared in advance). Also have some foods that typically are eaten with this food product (for example, sauces that the fufu is dipped into), and some plates.

	Step 3: Ask for a few volunteers to come up and wash their hands with soap and then help themselves to the food. Get them to sit in front of the group and eat the food.<br><br>Step 4: Make small talk with the volunteers as they eat. How is the food? Does it taste like it was made well? Do they think the person who made it washed their hands with soap? Why? Why not? Can they be sure? Addressing the audience, ask what did you notice about the way that the volunteers ate? How much were their hands involved in the eating process? Point out to the group how much contact the volunteers hands had with the food. Just as well they all washed their hands thoroughly before eating.<br><br>Step 5: Explain that sometimes we are busy looking after children or doing other household tasks and this may cause us to wash our hands with soap. It doesn't take much to contaminate hands and you can easily pick up germs from common surfaces in the household.<br><br>Step 6: Place some food dye on a plate. 'Let's imagine that this dye represents some germs on a surface. Perhaps it's the toilet door or the floor that I touched. Of course, in real life germs are invisible so we often think that or hands are clean even when they are actually dirty'. Dip your hand in the dye, ensuring quite a lot is on your hand. Now eat some of the traditional food in the same manner you observed the volunteers do (but a little more exaggerated). You should notice that the dye changes the colour of food, contaminating the whole thing.<br><br>Step 7: Take time to show the contamination to the audience. Explain to the group that in real life we often cannot see the germs on our hands. 'Handwashing with soap before cooking and eating is the only way we can be sure that we are not feeding our families contaminated food'.
Closing content	Watch this video: https://www.youtube.com/watch?v=w2qRcMTstzc from Ghana for inspiration on how to do this activity.
Tips	• Involve children: Children are likely to be less self-conscious about eating in front of a group and they are also likely to be messier eaters. So when asking for volunteers choose a few children aged 10 and up.<br>• Using food colouring (dye): Note that the food dye may temporarily discolour hands after doing this activity, but it should not cause any harmful effects and it will wash off eventually. It is fine to eat food with the dye on it.<br>• Plan community meetings: Community meetings are normally a great way of reaching lots of people. However, in some situations such as a disease outbreak (e.g. cholera or Ebola), or when working in unsafe areas, they should be avoided or planned very carefully so that no one is put at risk by attending. Make sure to take time to assess whether large community meetings might put your populations at risk. Remember that you may have to do multiple community events if you want to reach everyone. You may also have to make special efforts to ensure everyone is invited (for example, people with disabilities and older people often get left out of

	community meetings because it is harder for them to travel to the meeting location). Or you may need to create special types of events to ensure that people feel comfortable (for example, in some settings gender segregated events may be the cultural norm).<br>• Avoid creating fear: When altering risk perception, it is important to try to avoid just increasing fear. Fear has been found to increase handwashing behaviour in the short term but behaviour reverts as fear decreases. Fear can also have negative, unintended consequences on behaviour.

Activity name	Child Life Game
Cost	Inexpensive
Difficulty	Moderately difficult
Delivery time	45 minutes
Short description	Do a participatory play at a community event to show how the lives of two children can be dramatically changed by small moments of their lives.
Objectives	• Associate handwashing with being a good parent<br>• Associate handwashing with being wealthy, respected, educated and successful in life
Procurement required	None
Creative work required	Yes
What you need	Create two face-size masks. Both should feature the face of a child but one should be happy and the other should be sad. Both masks should depict children of a similar race, gender and age.
How to do it	Step 1: Organise a community meeting.<br><br>Step 2: Two facilitators are needed to tell the story, one will describe the situation affecting the first child while the other describes what happens to the second child.<br><br>Step 3: Ask for two volunteers. Get each volunteer to put on a mask.<br><br>Step 4: Explain to the crowd how the activity will work. Explain that whenever they hear something positive that will help the child to become strong, smart and successful, the appropropriate child can take a step forward. The crowd should clap each time they hear something positive so that the child knows to step forward. But if they hear something negative for their child's future, the volunteer should step back. They will know to step back because they will not hear you clapping.

	Step 5: Get the facilitators to read out their parts of the story below:<br><br>Facilitator 1: When the mother of child 1 is due to give birth she goes to a local clinic where the health workers always wash their hands with soap.<br><br>Facilitator 2: When the mother of child 2 is due to give birth she goes to the local clinic but finds that there is no water at the clinic and no soap so it's hard for the health workers to be hygienic.<br><br>Facilitator 1: Mother 1 is so proud of her child that she never wants to touch her without the cleanest of hands. She makes sure she washes her hands with soap every time she breastfeeds or feeds her child.<br><br>Facilitator 2: Mother 2 knows she should wash her hands with soap before feeding her child but often when her child cries she just wants to give her food straight away to stop her from being upset. So she skips handwashing.<br><br>Facilitator 1: Child 1 grows up in a simple house but it is a house that is always neat. Her mother taught her that if you enter the house after a hard day at work or school the first thing you need to do is wash your hands with soap. That way the dirt from outside stays outside!<br><br>Facilitator 2: Child 2 grows up in a simple house too but by the end of the day her parents are so tired that they just want to relax. No one ever reminds her to wash her hands with soap.<br><br>Facilitator 1: Child 1 lives in a house where the father built them a nice toilet with a handwashing facility outside. This makes it so easy for all the household members to always wash their hands.<br><br>Facilitator 2: Child 2 grows up in a family where there is no handwashing facility near the toilet. She thinks no one notices whether or not she washes her hands after defecation. But actually people do notice!<br><br>Facilitator 1: Child 1 thinks that her mother's cooking tastes amazing. One day she asked her mother what the secret was her mother said 'I always stop first to wash my hands with soap that way I always prepare you food with the cleanest of hands'.<br><br>Facilitator 2: Child 2 notices that her mother is often tired and distracted when she is making the food and her mother doesn't keep soap in the kitchen so it is not easy for her to wash her hands.<br><br>Facilitator 1: Child 1 does well at school. She is not naturally the brightest student but she has a secret for staying fresh and alert in class. Every morning and lunch time she washes her hands and splashes her face. That way she is ready to take on her day.

	Facilitator 2: Child 2 struggles at school and mainly looks forward to the break times when she and others rush to eat lunch. They are so hungry that they never bother washing their hands with soap.<br><br>Facilitator 1: Child 1 got the best marks in her school, grew up healthy and strong, and went on to study to be a doctor.<br><br>Facilitator 2: Child 2 struggled to get regular employment and often gets sick.<br><br>Step 6: Now look at where both children are. What do you think is the moral of this story? Why was Child 1 able to progress so much while child 2 got left behind? (give the audience a chance to respond).<br><br>Step 7: Explain that often it is the small actions we do in life, including good habits like handwashing with soap, which can make a big difference to how successful we are in the future.
Tips	• Prepare well: It is easy for this activity to become a bit chaotic. If this happens people might not understand the activity or see the point in it. To avoid this happening, make sure the facilitators practice the story beforehand and think about how they will manage the audience.<br><br>• Plan community meetings: Community meetings are normally a great way of reaching lots of people. However, in some situations such as a disease outbreak (e.g. cholera or Ebola), or when working in unsafe areas, they should be avoided or planned very carefully so that no one is put at risk by attending. Make sure to take time to assess whether large community meetings might put your populations at risk. Remember that you may have to do multiple community events if you want to reach everyone. You may also have to make special efforts to ensure everyone is invited (for example, people with disabilities and older people often get left out of community meetings because it is harder for them to travel to the meeting location). Or you may need to create special types of events to ensure that people feel comfortable (for example, in some settings gender segregated events may be the cultural norm).<br><br>• Avoid creating fear: When altering risk perception, it is important to try to avoid just increasing fear. Fear has been found to increase handwashing behaviour in the short term but behaviour reverts as fear decreases. Fear can also have negative unintended consequences on behaviour.<br><br>• Link this activity: This activity can work well when linked to other areas of your WASH programming. Try modifying the story to include other behaviours that you want to encourage in your community that contribute to making people healthy and strong.

Activity name	Social media tips

Cost	Inexpensive
Difficulty	Moderately difficult
Delivery time	4 months
Short description	Create a social media page or group where you can share the work you are doing and tips from community members who have improved their handwashing facilities.
Objectives	• Help make handwashing seem normative<br>• Provide social reward for people who have made changes to their handwashing facilities
Procurement required	None
Creative work required	Yes
What you need	• A camera
How to do it	Step 1: Use the results from the Wash'Em Touchpoints tool to identify which social media platform is most used in your area and set up a page or group. Choose a name for your group. Try to make this aspirational by using the results from the Wash'Em Motives activity. For example, Our strong community' or 'Families who care'. Add an icon for the group that depicts handwashing with soap.<br><br>Step 2: During household visits tell your population about the group and invite them to join.<br><br>Step 3: Where you see people making positive changes (e.g. building an innovative handwashing facility) you can ask to take a photo and share a quote on the social media page so that they become hygiene ambassadors for other people to admire. Alternatively, take a video of the person telling you about their handwashing facility. Get them to share their tips and information about the difference it has made to their lives.<br><br>Step 4: Encourage discussion on the group by posting questions or getting people to share photos of their own facilities. This way you can showcase how everyone is washing their hands and making positive change happen.
Tips	• Make sure you get permission: Make sure that any photos or videos that are shared on your social media page are shared with permission from the person/people in the photo. At the time of taking the photo make sure to clearly explain how it will be used on social media.<br><br>• Managing social media: Social media can allow you to interact with your target population remotely but in a very personal way. You can also get

	people to post photos of themselves participating in your program or doing a certain activity (like handwashing) so that the behaviour is seen as normative. However, social media has to be managed well in order to be effective. Make sure you dedicate some of your staff time for this purpose. Social media often creates a forum for discussion so it is important that your staff spend time moderating and responding to this discussion. This is particularly important if you plan to set up semi-private social media channels like Whatsapp. Social media can be used effectively to challenge rumours and misconceptions during crises. [Here](https://www.bbc.co.uk/news/world-africa-29573964) is an example from 2014 of a public health information Whatsapp channel around the West African Ebola outbreak.<br><br>• Link this activity: This activity can work best when linked to other areas of your WASH programming. Use your social media channel to promote all the different ways that your organisation is working with the community and share tips on other aspects of WASH such as menstrual hygiene, sanitation, water treatment, waste management, etc.

Activity name	Testimonies from survivors
Cost	Moderately expensive
Difficulty	Moderately difficult
Delivery time	3 days
Short description	This activity involves working with disease survivors in your community. Document their stories and experiences of contracting a disease and then share this with other people in the community via video or radio.
Objectives	• Help populations appreciate the full range of consequences that can occur if they or a family member gets sick<br>• Share hygiene tips that come from people with direct personal experience and a local perspective
Procurement required	None
Creative work required	Yes
What you need	• A video or audio recorder<br>• A way of sharing the stories - this could be via a) the local radio or TV channel, b) setting up a 'cinema' with a sheet and a projector, c) installing the videos on a tablet or phone that hygiene promoters can then take house–to–house or d) sharing them on social media.
Opening content	One of the best ways to challenge people's misconceptions is for them to hear real experiences from people who are like them.

How to do it	Step 1: Decide whether you will use videos or audio recordings, or both.<br><br>Step 2: Identify families who are survivors—meaning that someone in the family recently had serious diarrhoea/cholera/Ebola (whichever disease you are focused on). The quickest way of doing this may be to work with your local health centre.<br><br>Step 3: Ask if they are willing to become a hygiene ambassador and share their story with others. Explain to them how you plan to use their story.<br><br>Step 4: If they agree, conduct a recorded interview with them. Ask them to describe the following:<br>• What happened when they/their child got sick?<br>• How did they feel at this time?<br>• How did getting sick affect their social, work, and family life?<br>• How did it affect their productivity or income?<br>• What did they learn from the experience?<br>• What are they doing differently now to prevent anyone else in their family getting sick?<br>• How has their handwashing behaviour changed?<br><br>Step 5: Depending on the interview you may need to edit it to make it shorter. Final versions shouldn't be longer than 3 minutes.<br><br>Step 6: When sharing the stories, make sure to explain that these are people from their community. "Just like you, they thought sickness wouldn't seriously affect them or their families. They thought they were protecting their family as best as they could, but the experience has taught them that they could do more. And so now they do."
Tips	• Avoid creating fear: When altering risk perception, it is important to try to avoid just increasing fear. Fear has been found to increase handwashing behaviour in the short term, but behaviour reverts as fear decreases. Fear can also have negative, unintended consequences on behaviour.<br><br>• Highlight positive actions: It is important that these stories don't accidentally create stigma around the family. Make sure that the individuals are framed as people that they community should look up to and admire because they are now taking action to prevent further illness. Where possible, make sure that specific examples of positive actions are given.

Activity name	Can you smell the truth?
Cost	Inexpensive
Difficulty	Easy

Delivery time	30 minutes
Short description	Use a blindfold test to demonstrate how hands washed with soap smell good, while hands 'washed' without using soap do not.
Objectives	• Help people realise that soap is the magic ingredient that gets hands really clean<br>• Help people to realise that handwashing with water alone will not get hands truly clean
Procurement required	Yes
Creative work required	None
What you need	• Fish oil (as smelly as possible)<br>• Blindfold or scarf
Opening content	Markup text: Optional opening content
How to do it	Step 1: Organise a community event.<br><br>Step 2: Ask for three volunteers. Request for one of these people to be blindfolded. Their job is going to be to smell out which person has truly clean hands.<br><br>Step 3: Ask the other two volunteers to rub fish oil into their hands.<br><br>Step 4: Invite these volunteers to go up to other members of the audience and for the audience members to smell their hands. They should find the smell quite disgusting.<br><br>Step 5: Now get one of the volunteers to wash off the fish oil with water only. Get the other volunteer to wash their hands with water and soap. Now request the blindfolded volunteer to smell the hands of the other two volunteers. Can they tell which person used soap?<br><br>Step 6: Ask the blindfolded person if they would feel happy shaking hands with the volunteer who used soap. Presumably they would. Ask if they would feel happy shaking hands with the volunteer who only used water. Presumably they would be more reluctant.<br><br>Step 7: Remind people that only by using soap do our hands become truly clean. Make sure you are never caught out shaking hands with a smelly hand, by always using soap.
Tips	• Plan community meetings: Community meetings are normally a great way of reaching lots of people. However, in some situations such as a disease outbreak (e.g. cholera or Ebola), or when working in unsafe areas, they should be avoided or planned very carefully so that no one is

	put at risk by attending. Make sure to take time to assess whether large community meetings might put your populations at risk. Remember that you may have to do multiple community events if you want to reach everyone. You may also have to make special efforts to ensure everyone is invited (for example, people with disabilities and older people often get left out of community meetings because it is harder for them to travel to the meeting location). Or you may need to create special types of events to ensure that people feel comfortable (for example, in some settings gender segregated events may be the cultural norm).

Activity name	Don't miss out on the experience
Cost	Moderately expensive
Core	Complementary activity
Difficulty	Easy
Delivery time	2 days
Short description	This activity involves creating a sign or mural in your community which highlights how great people feel after handwashing with soap.
Objectives	• Increase the association between handwashing and feeling comfortable<br>• Help people realise that there are lots of non-health benefits to handwashing
Procurement required	None
Creative work required	Yes
What you need	• Coloured paint or a printed banner
How to do it	Step 1: Visit households and get people to tell you how they feel immediately after they wash their hands with soap. Get them to describe the feeling in just one word. Once you have asked this question to about 30 people, create a word cloud with the results.<br><br>Step 2: There are two ways of displaying your word cloud. The first option is find a prominent wall in your community and work with an artist to paint the words on the wall. The second option is to use an online programme such as this one: https://wordart.com to create the word cloud and then print this as a large banner or mural. When you design the mural or banner make sure it features all of the descriptive words and then a question: 'What were all these people describing?'. Avoid putting the answer on the poster. Instead, direct them to look on the other side of the billboard for example, or on the wall around the corner from the mural. This will allow you to build

	curiosity. On the other side of the billboard or wall, write ‘Handwashing with soap. Don't miss out on the experience.’
Tips	Think about the location: Take time to select the best place to hang your banner or paint your mural. Use the following criteria to help you:<br>• Where do people gather or spend time waiting?<br>• Where will it be seen by the most people?<br>• Is there a space where the view is not obstructed?<br>• Where are people likely to look? (avoid putting things high up or low down)<br><br>Link this activity: This activity can work just as well for other areas of your WASH programming. For example, you could create a similar wall describing sanitation as opposed to open defecation, or describing drinking treated water compared to untreated water (if these are issues in your community).

Activity name	Watching eyes
Cost	Inexpensive
Difficulty	Easy
Delivery time	15 minutes
Short description	Create stickers with a picture of eyes on them. Place the stickers above handwashing facilities to make people feel like others are noticing whether or not they wash their hands with soap.
Objectives	• Help people to realise that others do pay attention to whether or not they wash their hands with soap.
Procurement required	None
Creative work required	Yes
What you need	• Create stickers with eyes on them
Opening content	People are more likely to wash their hands if they think their behaviour will be noticed by others. Studies have shown that even just a picture of eyes near a handwashing facility can make people wash their hands 12% more. See this study for more info: https://onlinelibrary.wiley.com/doi/abs/10.1111/jasp.12501
How to do it	Step 1: Create stickers with an image of two eyes. You can add a message, for example: ‘People pay attention to whether you wash your hands’.<br><br>Step 2: During household visits, work with people to put up the stickers above their handwashing facility. Explain that these eyes will serve as a reminder that people pay attention to whether or not you wash your hands.
